# Association between total protein intake and low muscle mass in Korean adults

**DOI:** 10.1186/s12877-022-03019-1

**Published:** 2022-04-11

**Authors:** Youn Huh, Ki Young Son

**Affiliations:** 1 Department of Family Medicine, Uijeongbu Eulji Medical Center, Eulji Unversity, Daejeon, Gyeonggi-do Republic of Korea; 2 Department of Family Medicine, Asan Medical Center, University of Ulsan College of Medicine, Seoul, Republic of Korea

**Keywords:** Protein intake, Low muscle mass, Weight-adjusted low muscle mass, Korean, Adults

## Abstract

**Background:**

Adults with low muscle mass have a poor prognosis. Studies that examined the association between total protein intake and low muscle mass among adults are limited. Thus, we investigated the association between total protein intake and low muscle mass among Korean adults aged ≥19 years.

**Methods:**

We included 15,995 adults (6528 male and 9467 female) aged ≥19 years from the Korea National Health and Nutrition Examination Surveys (2008–2011). We divided the participants into groups according to protein intake quartile: Q1, Q2, Q3 and Q4 groups. The odds ratios (ORs) and 95% confidence intervals (CIs) of low muscle mass according to protein intake were analysed via multivariable logistic regression analysis. Stratified analyses according to sex, age and comorbidities were also performed.

**Results:**

Of the participants, 3.8% had weight-adjusted low muscle mass. The prevalence rates of low muscle mass were 1.5, 3.0, 3.9 and 7.2% in the Q4, Q3, Q2 and Q1 groups, respectively (*p* < 0.001). Compared with the Q4 group, the Q1 group had the highest ORs for low muscle mass, followed by the Q2 and Q3 groups (Model 5; OR, 95% CI: 2.03, 1.36–3.02 for Q3; 2.44, 1.64–3.61 for Q2; and 4.32, 2.89–6.45 for Q4) after adjusting for confounding variables (p for trend < 0.001). The associations between protein intake and low muscle mass were stronger in younger individuals, men, individuals without hypertension, those with diabetes mellitus and those without dyslipidemia.

**Conclusions:**

The prevalence of low muscle mass in Korean adults significantly increased with lower protein intake. Nutrition education for proper protein intake is also important for adults.

**Trial registration:**

Retrospectively registered.

## Background

The International Working Group on Sarcopenia stated that sarcopenia is associated with muscle mass loss alone or in conjunction with increased fat mass [1]. The prevalence of this condition defined by European Working Group on Sarcopenia in Older People (EWGSOP), 2010 at worldwide, is estimated to be more than 50 million worldwide, but this is expected to increase by four times in 2040 [2]. The prevalence of low muscle mass was 9.7% in men and 11.8% in women among Korean elderly people [3]. Low muscle mass increases the risk of fractures and is associated with serious medical outcomes in terms of frailty, disability, economic burden and death [4, 5]. Moreover, one study showed that low muscle mass decreased health-related quality of life, such as self-care, usual activities, anxiety and depression, for elderly Korean population [6]. Although muscle mass is not exactly a predictor of physical performance, low muscle mass is significantly correlated with sarcopenia. Low muscle mass could cause insulin resistance, diabetes mellitus, and metabolic syndrome, because skeletal muscles are sites for glucose uptake and deposition at normal fasting glucose [7–9]. In particular, low muscle mass in young is associated with mortality, metabolic syndrome, and sarcopenia [10–12].

Several factors may contribute to low muscle mass. Risk factors for low muscle mass include age, genetics, protein intake, physical inactivity [13], neurodegenerative disease, hormone deficiency and inflammation [14]. In particular, as low muscle mass is precipitated by a negative muscle protein balance [15], adequate protein intake is theoretically important for maintaining muscle mass. Insufficient protein intake increases the rate of muscle mass [16], grip strength [17], walking speed [18] and weight loss [19].

Some studies have shown that individuals with greater protein intake have less low muscle mass [16, 20, 21]. However, most of these studies were conducted in a limited population, such as elderly or postmenopausal women. Only a few cross-sectional studies have examined the association between total protein intake and low muscle mass among the general population, including younger adults. In particular, their association in terms of age, sex and chronic diseases, such as hypertension, diabetes mellitus and dyslipidemia, has not been fully investigated.

Therefore, we investigated the association between total protein intake and low muscle mass among Korean adults aged ≥19 years using the data from the Korea National Health and Nutrition Examination Survey (KNHANES), which was collected between 2008 and 2011. Furthermore, stratified analyses according to sex, age and comorbidities, such as hypertension, diabetes mellitus and dyslipidemia, were also performed.

## Methods

### Data source and study participants

We performed a cross-sectional study in which we analysed the data on individuals who participated in the second and third year of the 4th KNHANES (2008–2009) and the first and second year of the 5th KNHANES (2010–2011), because information about the appendicular skeletal muscle mass was only available during this period. Our study is a secondary analysis. The KNHANES comprises a health interview as well as nutrition and health examination surveys. It provides data on demographic characteristics, health behaviours, nutrition survey and health status collected through personal interviews, data obtained from physical examinations, as well as blood sampling performed in mobile examination centres. A stratified, multistage probability sampling design was used to select household units that participated in the survey.

We initially selected a total of 37,753 individuals who participated in the KNHANES between 2008 and 2011. Among them, we excluded data on adults aged ≤18 years (*n* = 9376). We excluded individuals who had any missing variables, such as appendicular skeletal muscle mass (*n* = 12,382). Finally, the data of 15,995 adults (6528 male and 9467 female) was analysed. The KNHANES was approved by the Institutional Review Board of Korea Centers for Disease Control and Prevention (IRB No: 2008-04EXP-01-C, 2009-01CON-03-2C, 2010-02CON-21-C and 2011-02CON-06-C) and the requirement for informed consent was waived because anonymous and de-identified information was used. Furthermore, our study adhered to the principles of the Declaration of Helsinki.

### Definition of protein intake

Nutrition survey of the KNHANES included the type and amount of food that participants ate 1 day before the survey. As a criterion for evaluating nutrient intake, the Korean nutrient intake standards were used. Protein intake was also investigated in the same way. In our study, protein intake was estimated as the amount of protein (g) divided by body weight. We divided the participants into groups according to protein intake quartile: Q1, Q2, Q3 and Q4 groups. The cutoff protein intake levels were 0.80, 1.08 and 1.49 g/kg/day in the male participants and 0.67, 0.93 and 1.28 g/kg/day in the female participants. Inadequate protein intake was defined as less than 0.8 g/kg/day according to the adequacy of the recommended dietary allowances (RDA) for protein [22]. In older adults, protein intake was suggested to be 1.0 g/kg/day or more, and this analysis was also conducted [23].

### Definition of low muscle mass

In KNHANES, dual-energy X-ray absorptiometry (Discovery-W; Hologic Inc., Waltham, MA, USA) was used to measure body composition. Appendicular skeletal muscle mass (ASM) was calculated as the sum of skeletal muscle mass for both arms and legs, based on the assumption that all fat-free and bone-free tissues are skeletal muscles [24]. The low muscle mass index was calculated as ASM divided by body weight (%) [25]. Since one study showed that ASM divided by body weight was associated with the other health outcomes than ASM divided by height^2^ [26], we used ASM adjusted by body weight. Low muscle mass was defined as a muscle mass index of > 2 standard deviations below the sex-specific mean for healthy individuals aged 20–39 years (reference group of the muscle mass index) [27]. The reference group of the muscle mass index included participants aged 20–39 years (2254 male and 3041 female), excluding those with a history of hypertension, dyslipidemia, cerebral infarction (stroke), myocardial infarction, angina, osteoarthritis, rheumatoid arthritis, osteoporosis, tuberculosis, asthma, allergic rhinitis, depression, renal failure, atopic dermatitis, diabetes mellitus, thyroid disease, any cancer, hepatitis and cirrhosis. The cutoff low muscle mass index levels were 27.28% in the male participants and 21.35% in the female participants for the definition of low muscle mass.

### Covariates

Age was divided into three groups: 19–39, 40–64 and ≥ 65 years. The monthly household income level was divided into two groups: the lowest quartile group and the second lowest to highest quartile group. Educational level was categorised into quartile groups: ≤6, 7–9, 10–12, ≥13 years. People who had smoked at least 100 cigarettes in their lifetime and continued smoking at the time of the survey were defined as current smokers. Individuals who had smoked at least 100 cigarettes in their lifetime and stopped smoking at the time of the survey were defined as ex-smokers, whereas those who had not smoked at least 100 cigarettes were defined as non-smokers. Risky alcohol consumption was defined as the consumption of an average of ≥5 and ≥ 3 glasses of soju (Korean liquor) per occasion by men and women during the past 30 days, respectively [28]. Exercise levels were determined by the question, ‘How many days in a week do you engage in 60 minutes of physical activity that increases your heart rate more than usual or causes you to become breathless?’, in which the participants could choose to answer either ‘0 to 4 days’ or ‘5 to 7 days’.

Height, weight and waist circumference (WC) were measured, and body mass index (BMI; kg/m^2^) was calculated as weight divided by height squared. The total energy intake was estimated based on the KNHANES nutrition survey using the 24-h recall method. The daily total calorie requirements were calculated by multiplying the standard weight by 35 kcal. The standard weight of men was determined as height (m^2^) × 22, whereas that of women was calculated as height (m^2^) × 21 [29]. An energy intake exceeding 120% of the total energy requirement was defined as overconsumption of calories.

Participants without a doctor’s diagnosis of hypertension, diabetes mellitus and dyslipidemia were regarded as not having them. In contrast, participants who self-reported having hypertension, diabetes mellitus and dyslipidemia or had a doctor’s diagnosis for these conditions were defined as having them.

### Statistical analyses

We combined data of KNHANES from 2008 to 2011 based on the raw data analysis guidelines of the KNHANES. Moreover, based on the complex sample design, we conducted all analyses by assigning a dispersed stratification estimation, stratification variables and weighted sample values. Continuous variables, such as BMI, low muscle mass index and nutrition, were analysed using the general linear model and presented as the mean and standard error. The categorical variables are presented as ratios and standard errors which were analysed using the chi-square test per period. Furthermore, to determine the association between protein intake as the exposition variable and weight-adjusted low muscle mass as the response variable, we conducted a multivariable logistic regression analysis and calculated the odds ratio (OR) and 95% confidence intervals (CIs). Variables significantly found in Table 1 were classified according to demographic, health behaviors, and chronic diseases, respectively, and then each category was added and adjusted for analysis. Model 1 was unadjusted. Model 2 was adjusted for sex, age, household income and educational level. Model 3 was additionally adjusted for smoking status and physical activity. Model 4 was additionally adjusted for comorbidities, such as hypertension, diabetes mellitus and dyslipidemia. Model 5 was additionally adjusted for calorie overconsumption. Model 5 was adjusted with variables with significant differences in chi-square test. Protein intake included both quartiles (g/kg/d) and a dichotomic variable (inadequate or adequate). Moreover, stratified analyses according to sex, age and comorbidities were also performed. Statistical significance was set at *p* < 0.05. All analyses were performed using the SPSS ver. 24.0 (IBM Corp., Armonk, NY, USA).

**Table 1:** Estimation of the basic characteristics of the participants

	ASM/weight		*P*-value
	Normal *N* = 15,244	Low *N* = 751	
Age (years)			< 0.001
19–39	98.1 (0.3)	1.9 (0.3)	
40–64	96.1 (0.4)	3.9 (0.4)	
≥ 65	90.5 (0.7)	9.5 (0.7)	
Sex			0.001
Male	96.8 (0.3)	3.2 (0.3)	
Female	95.6 (0.3)	4.4 (0.3)	
Education (years)			< 0.001
< 6	92.5 (0.6)	7.5 (0.6)	
6–9	94.6 (0.8)	5.4 (0.8)	
9–12	97.1 (0.3)	2.9 (0.3)	
≥ 12	97.8 (0.3)	2.2 (0.3)	
Income			< 0.001
Lowest	93.3 (0.6)	6.7 (0.6)	
Others	96.8 (0.3)	3.2 (0.3)	
Smoking status			0.006
Never smoker	96.1 (0.3)	3.9 (0.3)	
Ex–smoker	94.6 (0.7)	5.4 (0.7)	
Current smoker	96.8 (0.4)	3.2 (0.4)	
Alcohol consumption			0.145
Risky alcohol	96.9 (0.5)	3.1 (0.5)	
Non-risky alcohol	96.1 (0.3)	3.9 (0.3)	
Physical activity			0.005
Yes	96.7 (0.3)	3.3 (0.3)	
No	95.6 (0.3)	4.4 (0.3)	
BMI (kg/m^2^)	23.45 ± 0.04	27.33 ± 0.24	< 0.001
Calorie (kcal/day)	2047.21 ± 10.89	1722.09 ± 40.89	< 0.001
Fat (%)	17.59 ± 0.12	15.60 ± 0.41	< 0.001
Dietary protein intake (g/kg/day)	1.17 ± 0.01	0.86 ± 0.02	< 0.001
Hypertension			< 0.001
No	97.3 (0.2)	2.7 (0.2)	
Yes	90.4 (0.8)	9.6 (0.8)	
Diabetes mellitus			< 0.001
No	96.5 (0.3)	3.5 (0.3)	
Yes	91.0 (1.0)	9.0 (1.0)	
Dyslipidemia			< 0.001
No	96.5 (0.3)	3.5 (0.3)	
Yes	93.0 (0.8)	7.0 (0.8)	

*Abbreviation*: *ASM* Appendicular skeletal muscle mass, *BMI* Body mass index

Data is presented as mean ± standard error or percentage (standard error)

## Results

### Baseline characteristics

Table 1 shows the baseline characteristics of the 15,995 individuals according to muscle mass. The mean ages were 49.5 ± 0.5 years for all individuals, 54.9 ± 0.9 years for low muscle mass group and 44.2 ± 0.3 years for normal muscle mass group. The proportion of low muscle mass increased with increasing age, from 1.9% in 19–39 years to 9.5% in ≥65 years. The prevalence of low muscle mass decreased with higher education and income levels. The low muscle mass participants showed the largest prevalence of ex-smokers and lack of physical activity. The calorie intake per day was 1722.09 kcal/day in the low muscle mass participants and 2047.21 kcal/day in the normal muscle mass participants. The low muscle mass participants had higher mean BMI value than the normal muscle mass participants. The low muscle mass participants had low fat and protein intake than the normal muscle mass participants. The prevalence of low muscle mass was higher in participants with hypertension, diabetes mellitus, and dyslipidemia than those without the diseases.

### Prevalence of weight-adjusted low muscle mass according to protein intake

**Fig. 1:**
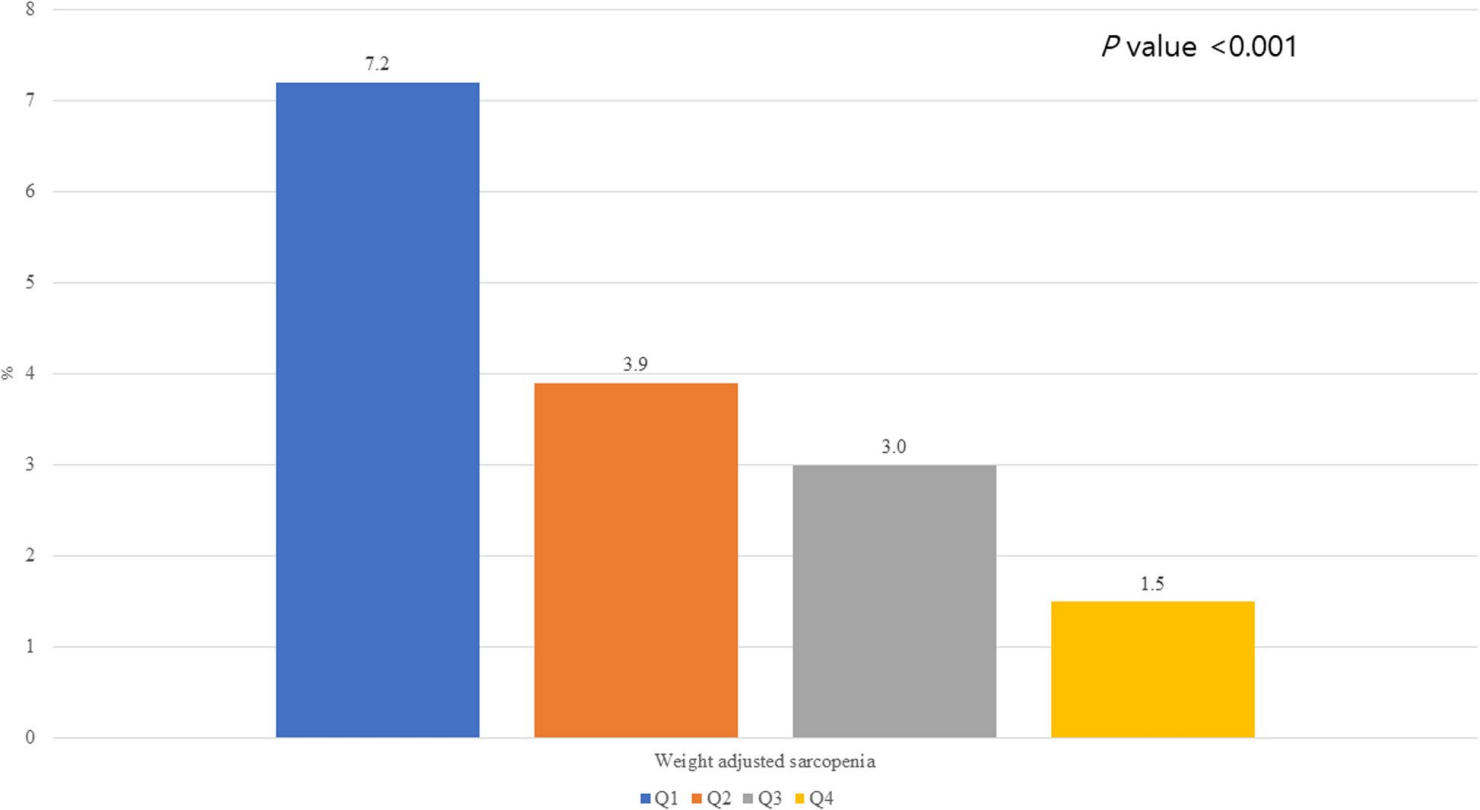
Prevalence of weight-adjusted low muscle mass according to protein intake quartile

Figure 1 presents the prevalence of weight-adjusted low muscle mass according to protein intake. Approximately 3.8% (*n* = 751) of the individuals had weight-adjusted low muscle mass. The prevalence of low muscle mass was 1.5% in the Q4 group, 3.0% in the Q3 of group, 3.9% in the Q2 group and 7.2% in the Q1 group (*p* < 0.001).

### Association between weight-adjusted low muscle mass and protein intake

Table 2 shows the multivariate analysis of the association between weight-adjusted low muscle mass and protein intake. Model 1 was crude model. After adjusting for confounding variables, the Q1 group had the highest ORs for low muscle mass, followed by the Q2, Q3 and Q4 groups. Compared with the Q4 group, the Q1, Q2 and Q3 groups had higher ORs for low muscle mass by 4.3 times (4.32 [95% CI, 2.89–6.45]), 2.4 times (2.44 [95% CI, 1.64–3.61]) and 2.0 times (2.03 [95% CI, 1.36–3.02]) after adjusting for confounding variables (Model 5), respectively (p for trend < 0.001). Compared with the adequate protein intake, the inadequate protein intake had higher ORs for low muscle mass by 1.9 times (Model 5; OR, 95% CI: 1.90, 1.52–2.37).

**Table 2:** Association between protein intake and weight-adjusted low muscle mass

	Model 1	Model 2	Model 3	Model 4	Model 5
	OR (95% CI)	OR (95% CI)	OR (95% CI)	OR (95% CI)	OR (95% CI)
Protein intake quartile (g/kg)					
Q1	4.96 (3.48–7.08)	3.75 (2.61–5.40)	3.80 (2.64–5.46)	3.60 (2.49–5.21)	4.32 (2.89–6.45)
Q2	2.61 (1.84–3.71)	2.12 (1.49–3.02)	2.13 (1.50–3.04)	2.07 (1.45–2.95)	2.44 (1.64–3.61)
Q3	2.02 (1.35–3.01)	1.83 (1.22–2.73)	1.84 (1.23–2.74)	1.82 (1.22–2.72)	2.03 (1.36–3.02)
Q4	1	1	1	1	1
*P* for trend	< 0.001	< 0.001	< 0.001	< 0.001	< 0.001
Nagelkerke	0.040	0.078	0.082	0.099	0.099
Protein intake					
Inadequate	2.54 (2.09–3.08)	2.03 (1.65–2.50)	2.04 (1.66–2.52)	1.95 (1.58–2.42)	1.90 (1.52–2.37)
Adequate	1	1	1	1	1
Nagelkerke	0.028	0.068	0.072	0.089	0.089

*Abbreviation*: *OR* Odds ratio, *CI* Confidence interval

Model 1 was not adjusted

Model 2 was adjusted for age, sex, household income and education

Model 3 was adjusted for age, sex, household income, education, smoking status, and physical activity

Model 4 was adjusted for age, sex, household income, education, smoking status, physical activity, hypertension, diabetes mellitus and dyslipidemia

Model 5 was adjusted for age, sex, household income, education, smoking status, physical activity, hypertension, diabetes mellitus, dyslipidemia, and overconsumption of calories

In older adults, compared to protein intake of 1.0 g/kg/day or more, protein intake of less than 1.0 g/kg/day had higher ORs for low muscle mass by 1.7 times after adjusting for confounding variables (Model 5; OR, 95% CI: 1.69, 1.15–2.48).

### Risk of weight-adjusted low muscle mass according to protein intake in the subgroups

Table 3 shows the results of the stratified analyses according to sex, age and comorbidities. In individuals aged 19–39 years, the OR increased by 6.1 times (6.06) [95% CI, 2.42–15.16]) in the Q1 group compared with the Q4 group, whereas the Q2 and Q3 groups did not show significant differences. Individuals without hypertension, dyslipidemia and diabetes mellitus showed stronger associations between protein intake and weight-adjusted low muscle mass compared with the other subgroups. In comparison with the Q4 group, the protein intake of the Q2 and Q3 group among individuals with hypertension and dyslipidemia was not significantly associated with weight-adjusted low muscle mass.

**Table 3:** Stratified analyses

	Protein intake (g/kg)			
	Q1	Q2	Q3	Q4
	OR (95% CI)	OR (95% CI)	OR (95% CI)	OR (95% CI)
Age (years)				
19–39 (*n* = 86)	6.06 (2.42–15.16)	2.33 (0.79–6.93)	2.46 (0.99–6.13)	1
40–64 (*n* = 307)	4.56 (2.66–7.82)	2.44 (1.50–3.98)	1.79 (1.10–2.92)	1
≥65 (*n* = 358)	2.81 (1.53–5.16)	2.09 (1.11–3.93)	2.02 (1.13–3.61)	1
Sex				
Male (*n* = 278)	6.49 (3.20–13.14)	3.16 (1.52–6.59)	2.83 (1.37–5.84)	1
Female (*n* = 473)	3.24 (2.05–5.13)	2.06 (1.32–3.21)	1.62 (1.06–2.50)	1
Hypertension				
(−) (*n* = 385)	6.02 (3.45–10.49)	2.91 (1.67–5.07)	2.35 (1.35–4.07)	1
(+) (*n* = 366)	2.35 (1.31–4.19)	1.63 (0.91–2.91)	1.54 (0.89–2.68)	1
Diabetes mellitus				
(−) (*n* = 615)	3.94 (2.61–5.95)	2.15 (1.42–3.25)	1.89 (1.25–2.85)	1
(+) (*n* = 136)	11.73 (3.09–44.56)	7.99 (2.02–31.61)	4.51 (1.23–16.52)	1
Dyslipidemia				
(−) (*n* = 632)	4.39 (2.85–6.75)	2.62 (1.70–4.05)	2.23 (1.43–3.47)	1
(+) (*n* = 119)	3.50 (1.41–8.72)	1.32 (0.53–3.30)	0.93 (0.36–2.39)	1

*Abbreviation*: *OR* Odds ratio, *CI* Confidence interval

The results were calculated via multivariable logistic regression analysis after adjustment for age, sex, household income, education, smoking status, physical activity, hypertension, diabetes mellitus, dyslipidemia, and overconsumption of calories

## Discussion

In this study, we investigated the associations between total protein intake and weight-adjusted protein among Korean adults aged ≥19 years. Compared with the Q4 group, the ORs for low muscle mass significantly increased in the Q1 group, followed by the Q2 and Q3 groups. In particular, the OR increased by six times in adults aged 19–39 years and by 12 times in those with diabetes mellitus in the Q1 group compared with those in the Q4 group after adjusting for confounders. Our results suggest that inadequate protein intake may be a risk factor for low muscle mass among adults aged ≥19 years.

Previous studies showed that protein intake is associated with muscle mass in older adults. A study including 740 older Australians showed that dietary nutrients had association with muscle mass and protein intake affects muscle mass and the rate of muscle loss [20]. And a study on community-dwelling elderly adults in Taiwan showed that groups with diets having the lowest quartile of protein intake had an increased risk of low muscle mass compared with those with diets having the highest quartile [30]. Furthermore, a meta-analysis of 14 randomized controlled trials including 1424 middle-aged to older adults revealed that higher protein intake significantly increased appendicular muscle mass [31]. In the last study, appendicular muscle mass was measured by dual-energy X-ray absorptiometry and 8 including 457 individuals of 14 trials included the analysis. However, these studies included only a small sample size and were limited adults aged ≥45 years.

The mechanism by which a lower intake of protein is associated with a higher prevalence of low muscle mass can be explained by some theories. Inadequate protein intake induces upregulation of the negative control of proliferation and down-regulation of muscle stem cell proliferation [32]. In addition, dietary protein intake increases the skeletal muscle synthesis and decreases the muscle proteolysis in postprandial state [33]. As low muscle mass is increased by a negative muscle protein balance [14], adequate protein intake is crucial for the maintenance of muscle mass.

The RDA for protein is 0.8 g/kg/day for all individuals aged 19 years and older. The RDA estimated requirement is considered a minimal requirement based on the avoidance of negative nitrogen balance studies conducted primarily in healthy young men [22]. As shown in Table 2, the ORs of low muscle mass were increased in the inadequate protein intake group compared with the adequate protein intake group. The Society on Sarcopenia, Cachexia and Wasting Disease recommends a protein intake level of 1.0–1.5 g/kg/day to prevent and mitigate low muscle mass [34]. Many Korean adults aged ≥19 years failed to meet this level of protein intake, because the cutoff levels of the highest and third quartile of protein intake in the male participants were 1.49 and 1.08 g/kg/day, while those in the female participants were 1.28 and 0.93 g/kg/day. In addition, other studies showed that people with a consumption of protein above of 0.8 g/kg/day have better physical performance score than people consume less than 0.8 g/kg/day [35, 36]. Therefore, dietary protein intake is a key component to improve skeletal muscle, prevent sarcopenia and impaired physical performance and loss of functionality in aged people. Nutrition education is needed to ensure adequate protein intake among adults.

Even when stratified by age, sex and the participants’ health status (with or without hypertension, diabetes mellitus and dyslipidemia), the risk of low muscle mass was greatest in the Q1 group, followed by the Q2, Q3 and Q4 groups. The association between protein intake and low muscle mass was stronger in the younger age group. Although many factors contribute to the anabolic resistance of muscle protein synthesis in older adults, minimal differences could be observed in the muscle protein synthesis rates between young and older adults after protein ingestion [37]. The association between protein intake and low muscle mass was found to be stronger among men, which appears to be attributed to sex hormones [14]. One study showed that muscle protein anabolism is impaired in adults with hyperinsulinemia, which is a characteristic of type 2 diabetes mellitus [38]. Our study showed that the association between protein intake and low muscle mass was stronger in individuals with diabetes mellitus than in those without diabetes mellitus. The Q1 group of proteins always had the highest risk of low muscle mass, even in subgroups of healthy adults without hypertension and dyslipidemia. Therefore, adequate protein intake might help reduce the risk of low muscle mass, even in young adults, men, individuals without hypertension, those with diabetes mellitus and those without dyslipidemia.

Moreover, one study [11] showed that muscle strength, such as knee extension and handgrip, is associated with all-cause mortality and cardiovascular disease induced mortality among male adolescents. In addition, young adults with low muscle mass are associated with higher risk of metabolic syndrome without obesity [12]. The loss of muscle mass starts from 30s and induces in immobility and dependence in old age [39]. Therefore, low muscle mass in young age as important as low muscle maa in older age.

However, this study has several limitations. First, because of the nature of cross-sectional studies, the causal relationships between protein intake and low muscle mass among adults could not be explained. Second, although we used the most common definition of low muscle mass, we could not include other definitions. Third, because the health status and lifestyle of the participants were based on self-reported questionnaires, the data could be subject to recall bias. Furthermore, the 24-h recall method for calculating calorie intake did not lead to an overall average because of daily stark variations. Although we might have considered some of the factors that influenced the study outcomes, all confounding variables were not likely accounted for. Despite these limitations, the results of the present study indicated that inadequate protein intake may be a risk factor for low muscle mass among adults aged ≥19 years using a representative Korean sample. Thus, clear and actionable solutions are needed in the public health domain to mitigate the consequences of low muscle mass.

## Conclusion

The low muscle mass in Korean adults aged ≥19 years is associated with a lower intake of protein. Proper protein intake is necessary to prevent low muscle mass in adults. Nutrition education for proper protein intake is also important for adults aged ≥19 years, especially for individuals without chronic diseases, such as hypertension and dyslipidemia.

## Data Availability

All data underlying the authors’ findings in this study are freely available in Korea Centers for Disease Control and Prevention. If interested in requesting these data, please visit the following link for more information: https://knhanes.kdca.go.kr/knhanes/main.do

## Abbreviations

ASM: Appendicular skeletal muscle mass
BMI: Body mass index
CI: Confidence interval
KNHANES: Korea National Health and Nutrition Examination Survey
OR: Odds ratio
RDA: Recommended dietary allowances
WC: Waist circumference
